# Two-photon fluorescence lifetime imaging microscopy of NADH metabolism in HIV-1 infected cells and tissues

**DOI:** 10.3389/fimmu.2023.1213180

**Published:** 2023-08-16

**Authors:** Greg A. Snyder, Sameer Kumar, George K. Lewis, Krishanu Ray

**Affiliations:** ^1^ Division of Vaccine Research, Institute of Human Virology, University of Maryland School of Medicine, Baltimore, MD, United States; ^2^ Department of Microbiology and Immunology, University of Maryland School of Medicine, Baltimore, MD, United States; ^3^ Department of Biochemistry and Molecular Biology, University of Maryland School of Medicine, Baltimore, MD, United States

**Keywords:** NADH metabolism, HIV-1, infected cells and tissues, oxidative phosphorylation, two-photon fluorescence lifetime imaging microscopy

## Abstract

Rapid detection of microbial-induced cellular changes during the course of an infection is critical to understanding pathogenesis and immunological homeostasis. In the last two decades, fluorescence imaging has received significant attention for its ability to help characterize microbial induced cellular and tissue changes in *in vitro* and *in vivo* settings. However, most of these methods rely on the covalent conjugation of large exogenous probes and detection methods based on intensity-based imaging. Here, we report a quantitative, intrinsic, label-free, and minimally invasive method based on two-photon fluorescence lifetime (FLT) imaging microscopy (2p-FLIM) for imaging 1,4-dihydro-nicotinamide adenine dinucleotide (NADH) metabolism of virally infected cells and tissue sections. To better understand virally induced cellular and tissue changes in metabolism we have used 2p-FLIM to study differences in NADH intensity and fluorescence lifetimes in HIV-1 infected cells and tissues. Differences in NADH fluorescence lifetimes are associated with cellular changes in metabolism and changes in cellular metabolism are associated with HIV-1 infection. NADH is a critical co-enzyme and redox regulator and an essential biomarker in the metabolic processes. Label-free 2p-FLIM application and detection of NADH fluorescence using viral infection systems are in their infancy. In this study, the application of the 2p-FLIM assay and quantitative analyses of HIV-1 infected cells and tissue sections reveal increased fluorescence lifetime and higher enzyme-bound NADH fraction suggesting oxidative phosphorylation (OxPhos) compared to uninfected cells and tissues. 2p-FLIM measurements improve signal to background, fluorescence specificity, provide spatial and temporal resolution of intracellular structures, and thus, are suitable for quantitative studies of cellular functions and tissue morphology. Furthermore, 2p-FLIM allows distinguishing free and bound populations of NADH by their different fluorescence lifetimes within single infected cells. Accordingly, NADH fluorescence measurements of individual single cells should provide necessary insight into the heterogeneity of metabolic activity of infected cells. Implementing 2p-FLIM to viral infection systems measuring NADH fluorescence at the single or subcellular level within a tissue can provide visual evidence, localization, and information in a real-time diagnostic or therapeutic metabolic workflow.

## Introduction

The increasing prevalence of antimicrobial resistance, including seasonal, past, and present global viral infections and co-infections (HIV, Ebola, H1N1, Zika, and the recent COVID-19 pandemic) underscores an urgent medical and scientific need for improved and versatile imaging modalities of affected cells and tissues (1). Accordingly, there is an unmet scientific need for developing a rapid, quantitative, intrinsic, minimally invasive, and label-free imaging of virally infected cells and tissues. In particular, HIV-1-infected individuals have increased morbidity-related inflammation and metabolic dysfunction despite having well-controlled viral loads. To address this need, we have employed a quantitative, minimally invasive 2p-FLIM method to detect changes in NADH metabolism of HIV-1 infected host cells and tissues. NADH is used as a sensor for metabolic events (2). 2p-FLIM utilizes the autofluorescence of NADH and NADPH molecules to measure cellular metabolism changes in contrast to oxidized forms NAD+/NADP+, which are not fluorescent (3, 4). Fluorescence lifetime imaging allows quantifying free and protein-bound NAD(P)H independent of intensity (3, 5–8). Cytoplasmic and nuclear NAD+/NADH ratios are estimated at ~700:1 (2). Therefore, discrete alterations in cellular NAD+ are likely to be reflected as significant changes in NADH. Implementing 2p-FLIM for measuring NADH fluorescence of single infected cells and tissue can quantitatively image and assess cellular, subcellular, and tissue-specific localization during viral infection and affected surrounding tissue in a minimally invasive near real-time workflow.

Fluorescence imaging spectroscopy is a central technology used throughout analytical chemistry, clinical chemistry, drug discovery, proteomics, genomics, and biochemical research. Fluorescence detection is accomplished almost without exception using extrinsic fluorophores to label the biomolecules. Fluorescence has received significant attention in the last two decades for *in vitro* and *in vivo* imaging; however, most of these relied on bulky and hydrophobic exogenous probes and intensity-based imaging. In contrast, two-photon imaging excitation inherently illuminates a very small sample volume, improves axial resolution, clearly separates excitation and emission wavelengths, reduces photobleaching, and provides depth discrimination and good signal-to-noise ratio (9–13). Moreover, biological samples are more tolerant to > 750 nm near-infrared (NIR) illumination, and there is a reduction in autofluorescence and scattering. Accordingly, multiphoton excitation is becoming increasingly popular (14).

Standard methods used in biosciences are steady-state techniques based on analyzing the total fluorescence signal originating from the sample. FLIM is an advanced quantitative optical method valuable for biological and biomedical applications (15–22) and offers contrast on images corresponding to the fluorescence decay times. It is becoming more widely used for quantitative studies of cellular processes and biomedical applications, including tissue morphology and high-density protein arrays (7, 23–26). Fluorescence lifetime is an inherent parameter that depends on the molecular environment of the fluorescent probe but is independent of probe concentration, excitation intensity, and light scattering. The fluorescence decay rates reflect the molecular interactions of a probe with its biological environment. The fluorescence decay rates of the probe may be affected after its interaction with a target, thus the FLT of the probe in cells or tissues can reflect when the probe is free or bound to a target. Further, FLIM mitigates the background signals of cellular imaging as the lifetime of the fluorophore can be differentiated from the background signal. Moreover, in case of two-photon excitation, the fluorescence intensity is proportion to the square of the peak excitation intensity, which is highest at the focal plane, and thus the off-axis signal and background are reduced substantially as demonstrated by many studies over two decades (5, 12, 27). The spatial resolution of intracellular structures is possible in images that provide temporal and spatial information on changes in the FLT of fluorescently labeled components. The structural and biochemical processes can be observed and quantitatively analyzed. The image contrast in FLIM is not dependent on fluorophore concentration.

Our studies distinguish free and bound populations of NADH by their different fluorescence lifetimes (3, 5–8). Moreover, the fraction of NADH bound to protein has a higher intensity (2, 4). Here, we have extracted profiles of NADH binding within HIV-1 infected cells or isolated tissues. It is challenging to distinguish NADH and its phosphorylated form NADPH based on their overlapping spectral properties (13, 27); we refer to it as NADH for brevity. We expect 2p-FLIM methodology to be uniquely sensitive to cellular changes in NADH levels resulting from either pathogen-mediated perturbations or expected differences in glycolysis or respiration under aerobic and oxidative phosphorylation (6-NADH) versus anaerobic respiration (4NADH) or those resulting from metabolic disruptions, syndromes or diseases (1, 28). Microbial infections induce alterations in both host innate immune and metabolic (Glycolytic and OxPhos) signaling pathways (1, 29–32). NAD+ is released into the cytoplasm as a sensor of pathogen infection (33, 34). The ability to quickly and with limited intervention assess cells and tissue exhibiting changing or perturbed levels of NADH under various aerobic and anaerobic environmental, microbial, metabolic, and disease conditions represents a broad platform for diagnostic and therapeutic development.

**Figure 1:**
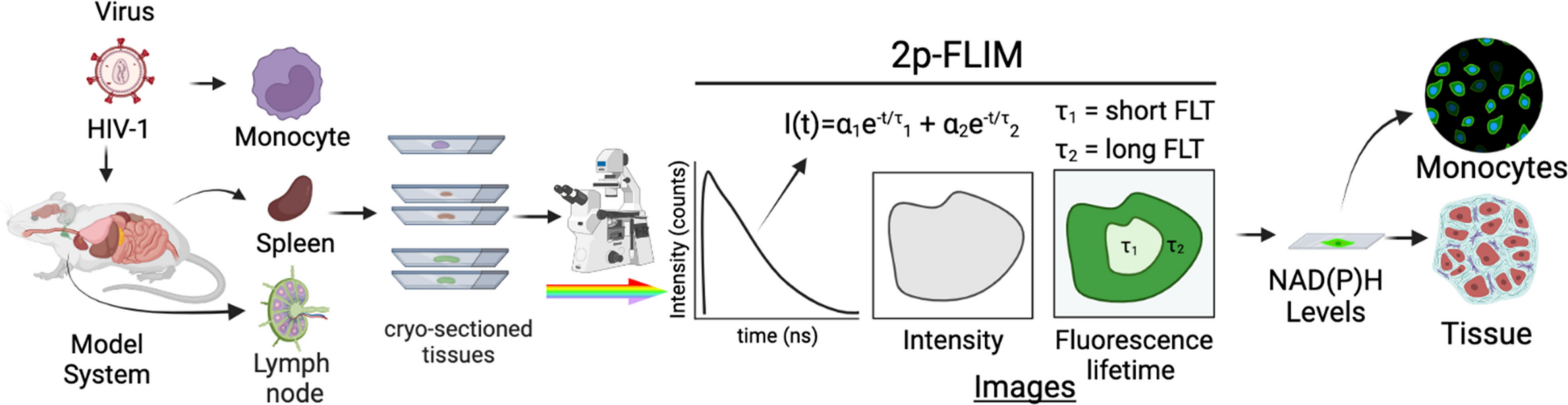
Experimental setup of label-free live 2p-FLIM imaging of NADH in HIV-1 infected cells and mouse tissue sections. HIV-1 infected cells and animal model organ tissue are cryo-sectioned and imaged using 2p-FLIM. 2p-FLIM analyses are used to image and quantitate NADH levels and lifetimes for infected cells and tissue types (see methods). The graphical illustration is generated using Biorender.

The label-free 2p-FLIM imaging technique at a single infected cell level will have broad applicability in understanding the metabolism in the innate microbial system (**Figure 1**). It will help elucidate the connection between HIV-1 infection and observed metabolism changes. The ability to quantitate and directly monitor NADH metabolism in real-time in HIV-1 infected cells and tissues offers a facile method for understanding microbial pathogenesis that can be applied to other infection models. Two different cell lines (THP-1 and MOLT-4/CCR5), four different types of HIV-1 viruses were used for this study: 1) HIV-1 BaL, a “tier 1b” virus which has a relatively “open” Env structure, more sensitive to neutralization by a wider variety of anti-envelope monoclonal antibodies, 2) A tier 2 envelope pseudovirus, HIV-1 JRFL which is hard to neutralize, 3) The transmitted/founder isolate AD-17 is replication-competent infectious molecular clone and express unmodified native trimers, and 4) An eGFP HIV-1 reporter virus that can infect cells by which infection (p24 content) and eGFP levels can be correlated. To complement the *in vitro* cellular studies, 2p-FLIM analyses approaches were applied to perform NADH metabolic imaging within infected tissues from spleen and lymph node of HIV-1 infected humanized NSG mice and compared with tissue sections from uninfected NSG mice. Overall, the label-free imaging approaches described in this paper have the distinctive potential to address biomedical research needs and technical problems that occur broadly across multiple biological systems or diseases.

## Materials and methods

### Cell lines and HIV-1 virions

Commercial cell lines THP-1 and MOLT-4/CCR5 obtained from ATCC are provided with certification that they are *Mycoplasma*-free. Stock cultures are grown up, and aliquots are frozen down to enable the use of the same cell lines throughout the study. The cell line was maintained in the respective cell-culture media at 37°C/5% CO_2_/125 rpm shaker.

### Construction of HIV-1/eGFP (pSF345-02-gfp)

To produce the R5 tropic HIV-1/eGFP pBR43IeG-nef+/EGFP (AIDS Research and Reagents Program #ARP-11100) was used as a parental plasmid construct. The pBR43IeG-nef+-EGFP V3-Env region was removed by digestion with StuI/BsaBI restriction enzymes (NEB, Ipswich, MA) following the treatment with Calf Intestinal (CIP) Alkaline Phosphatase (NEB, Ipswich, MA) to prevent self-ligation. Next, gel purification was performed to remove the pBR43IeG-nef+-EGFP V3 Env region. DNA synthesis (Blue Heron Bio, Bothell, WA) was used to recreate the excised pBR43IeG-nef+-EGFP V3-Env sequence containing a BaL V3 DNA sequence. Synthesized DNA was digested with StuI/BsaBI (NEB) and then gel purified. DNA fragments were ligated with T4 DNA ligase (NEB) and used to transform STBL2 cells (Invitrogen, Waltham, MA). Clones were screened by restriction mapping, (EcoRI/StuI, NEB) with two correct restriction digest pattern clones selected for sequence confirmation (CIBR, Baltimore, MD).

### HIV-1 virion production

HIV-1 JRFL pseudoviruses were generated by co-transfection of HEK293T cells with an Env-deficient HIV-1 backbone plasmid pNL4-3-ΔE-EGFP along with Env-expression plasmids (35, 36) and pCAGGS-JRFL (kindly provided by J. Binley, Torrey Pines Institute of Molecular Studies, San Diego, CA). Transfections were accomplished using FuGENE 6 (Roche, Indianapolis, IN) transfection reagent at a 3:1 reagent-DNA ratio. To produce the infectious molecular clone of HIV-1 BaL or transmitted/founder (T/F) HIV-1 AD17 virus (37) or HIV-1/eGFP, HEK293T cells were transfected with the BaL (obtained through the AIDS Research and Reference Reagent Program, Division of AIDS, NIAID) or AD17 plasmid (kindly provided by B. Hahn, University of Pennsylvania) or pSF345-02-gfp plasmid at a FuGENE-to-DNA ratio of 3:1. Virions-containing supernatant was harvested after three days, and concentrated about 10-fold by incubating with PEG-*it*™ virus precipitation solution (System Biosciences, Mountain View, CA) for 18 hours at 4°C as recommended by vendor. The antigen content of all virion preparations was quantified using p24 and gp120 antigen capture ELISAs. Infectivity was established using standardized procedures (38) and quantified as a function of TCID_50_ in TZM-bl cells. HIV-1 BaL and HIV-1 JRFL pseudoviruses with gp120 to p24 ratio of 1:10-1:50, and 200,000 – 500,000 TCID50/mL; HIV-1 AD17 T/F with gp120 to p24 ratio of 1:200, and 600,000 to 1,000,000 TCID50/mL; were used.

### HIV-1 infection assay

MOLT4/CCR5 cells were infected using MOI 1 of HIV-1/eGFP *via* spinoculation at 2000 rpm for 2 hours at 7°C. Cells were washed and seeded at a concentration of 2×10^6^/mL. After 48 hours, 10 µL of cell suspension was incubated in poly-lysine coated slides for 30 mins at room temperature and washed two times before imaging under a 2p-FLIM microscope.

### Evaluating viral infection on NADH metabolism in mouse tissues

To parallel the results of the *in vitro* studies in the previous section and complement the outcome allowing cross-comparisons of *in vivo* and *in vitro* data and the generation of a comprehensive picture of NADH metabolism in tissues, we have performed NADH fluorescence in HIV-1 infected humanized mouse tissues. Furthermore, HIV-infected tissues allow us to survey the host-pathogen interaction and determine the tissue level response to infection. Accordingly, the 2p-FLIM method investigated NADH metabolism in cryo-sectioned p24 confirmed HIV-1 infected tissues. NADH levels in HIV-infected mouse tissues (specifically spleen and lymph nodes) were quantified using 2p-FLIM and compared with the uninfected tissues. NSG mice reconstituted with human PBMCs (hu-PBMC-NSG-SGM3) (39) were used for the current study investigating HIV-1 infection in a humanized mouse model. These strains/models do not suffer significantly from graft versus host (GVH) effects. This is a robust humanized mouse model, which unavoidably enhances HIV replication *via* developing GVH immune activation.

### Humanized NSG mice tissue sectioning and staining

Fixed, frozen 7µm thick tissue sections of lymph-node and spleen from HIV-1 BaL-infected humanized NSG mice or controlled humanized NSG mice (39) were made on a Leica CM1860 UV - Cryostat. Cryo-sectioned tissues were mounted on a glass slide with Invitrogen prolong antifade reagent. HIV-1 p24 monoclonal antibody D45F tagged with FITC (Invitrogen catalog # MA1-7378) was used to stain the 7µm thick lymph node tissue section from HIV-1 infected mice.

### Microscope setup

A customized confocal microscope (based on ISS Q2 laser scanning nanoscope) with single-molecule detection sensitivity was used for performing 2p-FLIM. The excitation source is a pulsed femtosecond laser (Calmar, 780nm, 90 fsec pulse width, and 50MHz repetition rate) equipped with an ISS excitation power control unit. An incident wavelength of 780 nm was used for exciting NADH in cells or tissue samples (27). The excitation light was reflected by a dichroic mirror to a high-numerical-aperture (NA) water objective (60X; 1.2 NA) and focused onto the sample. The fluorescence was collected by single photon counting avalanche photodiodes (SPAD) through a dichroic beam splitter, Chroma short pass (750SP), and 460/55 bandpass filters, thus eliminating the scattered excitation light and collecting fluorescence from the NADH in the region of interest. Using the 780nm two-photon excitation, eGFP fluorescence intensity and lifetime were recorded in the 500 to 529nm spectral window with a narrow bandpass filter (514/30, Chroma). We used one excitation wavelength for simultaneous detection of both NADH and eGFP in two separate detection channels that included high quality bandpass emission filters without spectral bleed through. NAD(P)H fluorescence detection has been reported in the literature using 780nm excitation (3, 27). The imaging in our Q2 setup was performed with Galvo-controlled mirrors with related electronics and optics controlled through the 3X-DAC control card. The software module in ISS VistaVision for data acquisition and processing and the time-correlated single photon counting (TCSPC) module from Becker & Hickl (SPC-150) facilitate FLIM measurements and analyses. Each image included 512x512 pixels with a dwell time of 200 µsec for each pixel translating to an image acquisition time of ~53sec. The decay sampling resolution was 19.5 psec which was determined from the number of the decay time bins and the total time length of the decay. The FWHM of the measured IRF by directly recording the laser scatter light was ~ 300psec. The intensity decays were fitted with a bi-exponential model: I(t) = α_1_exp(-t/τ_1_) + α_2_exp(-t/τ_2_), where τ_1_ and τ_2_ are the short and long decay times; α_1_ and α_2_ are the amplitudes for the short and long decay times respectively (**Figure 1**). The sum of the amplitude fractions (α_1_ + α_2)_ in the observed biexponential are normalized to unity. The fractional contributions of short and long components to the steady-state intensity are denoted as f_1_=α_1_τ_1_/(α_1_τ_1_+α_2_τ_2_) and f_2_=α_2_τ_2_/(α_1_τ_1_+α_2_τ_2_). The average (intensity-weighted) lifetime is described by f_1_τ_1_+f_2_τ_2_. The values of FLTs (τ_1_ and τ_2_) were obtained using the ISS vistavision software with the deconvolution of instrument response function and nonlinear least-squares fitting. For the decay analyses we used the estimated IRF generated by the ISS VistaVision software. Three different fields of view per independent experiment were used to compute the average of different metrics. For the HIV-1 infected cell samples at least 20 cells were used for calculating various metrics.

All time-resolved fluorescence data were analyzed using ISS VistaVision software. TCSPC histograms of individual pixels are depicted as usual as the logarithm of the time resolved intensity as a function of time. TCSPC histograms were mostly fitted by a standard method to a bi-exponential decay model using the Marquardt algorithm, by minimizing the sum of the squares of differences between the measured and calculated values of time-dependent intensity and calculating a reduced χ^2^. Two components were usually chosen for NADH decay analyses because the raw data indicate there is more than one component (the decay lines aren’t linear), with multiple fluorescent lifetimes present. For determining the FLTs (τ_1,_ τ_2_, and average lifetime), pre-exponential factors α_1_, α_2_ and fractional contributions from each measurement, the regional average of the time-resolved fluorescence decays were fitted with a bi-exponential model as described before. However, the intensity decay for the eGFP channel could be fitted using a single-exponential decay model. The difference (residuals) between the actual measured intensity and the calculated value from the fit at that time point were evaluated. The standard deviations of the residuals were evaluated to assess the quality of the fit. A good fit is characterized by residuals that are mostly small (90% are less than 2 standard deviations) and randomly distributed around zero. The derived χ^2^ value is expected to be close to 1.0.

We have avoided the crosstalk between NADH and eGFP channels using high quality narrow bandpass emission filters (Chroma) which minimize the spectral overlap in the spectral region of 435-485nm for the detection of NADH signal and 500-529nm for the detection of fluorescence from eGFP. The quantum yield of eGFP is significantly (over 10-fold) higher than NADH (4, 40). Furthermore, fluorescence recorded in the detection channel using a high quality 514/30 bandpass emission filter showed a single exponential decay with a fluorescence lifetime of 2.6 nsec confirming that the fluorescence emanated from eGFP only in 500 to 529 nm spectral range (15). Moreover, detection of NADH fluorescence with a narrow bandpass filter in the spectral region of interest alleviated the issue of fluorescence contributions from other structural proteins (collagen or elastin) (4). We have avoided any emission spectral overlap between flavins and NADH using high quality narrow bandpass filters in the spectral region of 435-485nm for detection of NADH signal. For each experimental FLIM measurement with the HIV-infected tissue samples, we included control mouse tissue samples detected under identical settings (same excitation wavelength, incident laser power, detection emission filter, image acquisition parameters). We have used tissues from three HIV-1 BaL infected mice for FLIM studies, correspondingly tissues were collected from uninfected controlled mice. In all HIV infected tissue section, either from spleen or lymph nodes, we consistently observed higher NADH signals in infected tissues compared with the control mouse tissues. Furthermore, the quantum yield of collagen and elastin fluorescence is lower than NADH either free or protein bound form. The emission spectra of collagen (emission maximum at 400nm) and elastin (emission maximum at 415nm) are ~50nm blue-shifted compared to NADH emission spectra (4).

Statistical analysis of data was performed using GraphPad Prism 8.0 and Origin 2021 software. A two-tailed test evaluated the statistical differences between control and infected cells or tissues. A p-value of less than 0.05 was considered statistically significant.

## Results

2p-FLIM studies were performed to determine and characterize cellular metabolism utilizing the NADH fluorescence spectral window in HIV-1 virus-infected cells. The model systems for studying *in-vitro* viral infections in cell lines include HIV-1 pseudoviruses and infectious molecular clones (IMC) with THP-1 and MOLT-4/CCR5 cells. Quantitative 2p-FLIM analyses distinguish free and bound populations of NADH by their different fluorescence lifetimes within single infected cells. NADH fluorescence measurements in a single cell could provide necessary insight into the heterogeneity of metabolic activity in viral-infected cells. It is important to note that viruses do not have any NADH fluorescence alone. Accordingly, any changes in NADH signal level upon virus infection to the host cells could be related to the changes in metabolic activity in the infected cells. Separate vertical scale bars were used for each intensity image (kilo counts per sec, KCPS) and fluorescence lifetime image (nanosecond, nsec). A linear red-green-blue (RGB) colored scale bars indicating changes in fluorescence lifetimes (FLTs) from 0 to 5 nsec range are displayed for each FLIM image panel. The present study and experiments focus on determining the changes in protein bound NADH with metabolic perturbations (27) in HIV-1 infected cells and tissues. For clarity, the lifetime of free NADH (τ_1_) is not displayed as a FLIM image. However, average fluorescence lifetime images containing both fast and slow lifetime components are shown.

### Quantitative determination of NADH fluorescence in HIV-1 infected cells

The test panel includes HIV-1 virus phenotypes and neutralization sensitivities (tiers) (41, 42). We examined pseudoviruses expressing neutralization-resistant tier-2 CCR5-tropic HIV-1 JRFL, tier-1 HIV-1 BaL infectious molecular clones (IMC) (43–46), and transmitted/founder (T/F) (47, 48) HIV-1 subtype B AD17 viruses, which are replication-competent IMC and express unmodified, native trimers (45, 46). The pseudoviruses have only a single round of infection, whereas the T/F or IMC virions are replication-competent and have multiple rounds of infection. All virion preparations are characterized for Env protein content, p24 protein content, and viral RNA copies by in-house quantitative PCR. We emphasize that there is no further genetic or chemical manipulation of the envelopes on these viruses.

**Figure 2:**
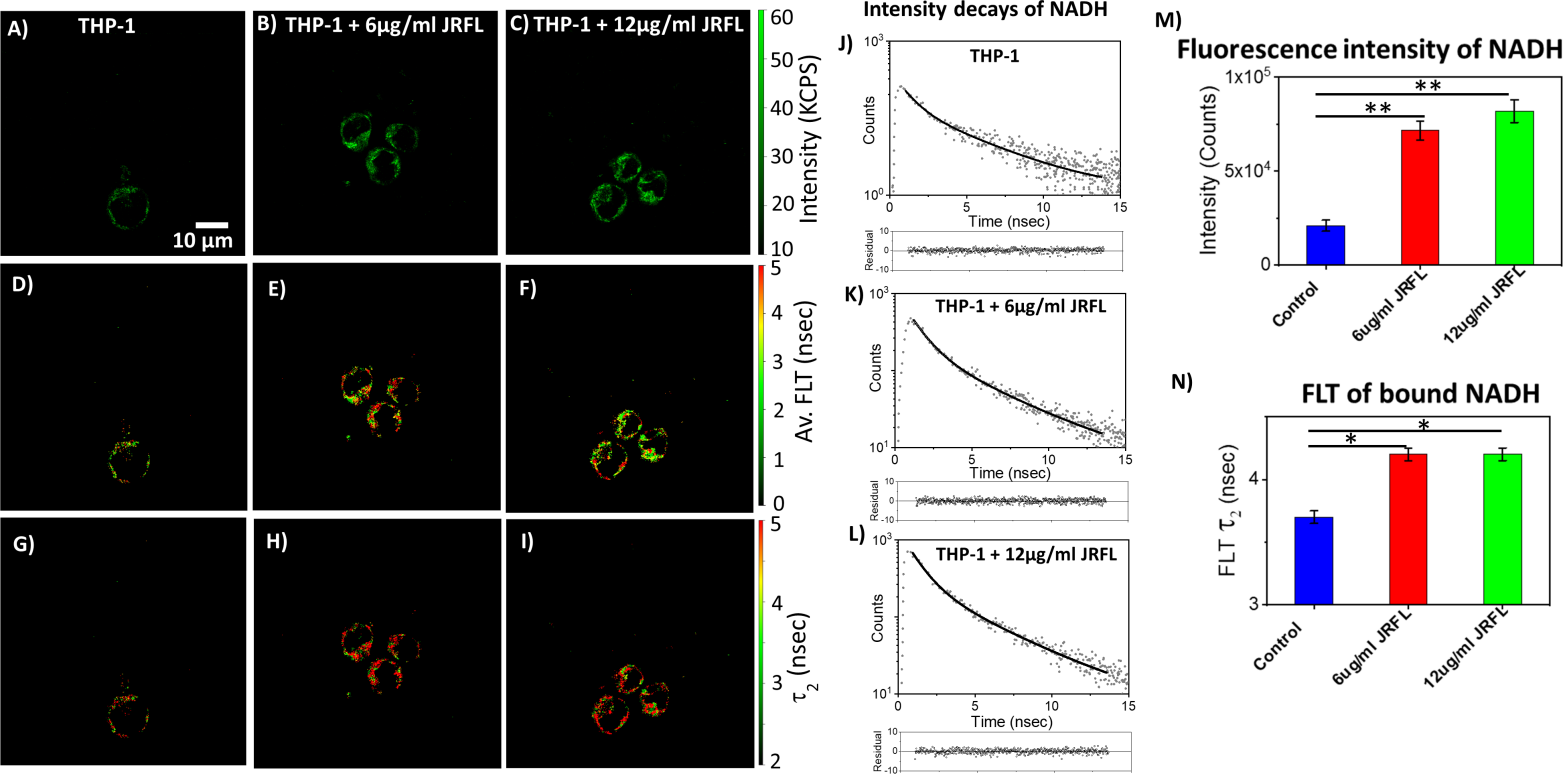
2p-FLIM identifies differences in NADH fluorescence intensity and lifetimes of infected cells. Label-free live 2p-FLIM imaging of NADH with THP-1 cells upon infection with HIV JRFL pseudovirions at different virion concentrations. Differences in NADH fluorescence intensity (kilo counts per second: KPCS) **(A–C)**, average FLT of NADH **(D–F)**, and bound NADH FLT **(G–I)** with THP-1cells infected with HIV JRFL pseudovirus compared to THP-1 control. **(J–L)** show the time-resolved intensity decays of NADH from uninfected and HIV JRFL pseudovirus (p24 values of 6µg/ml and 12µg/ml) infected cells. The dots represent the experimental data and solid line is the bi-exponential fit to the intensity decay (see methods). Residuals are plotted below each graph in **(J–L)** showing the residual values are randomly distributed around zero. Image intensity and bound form of NADH FLTs are quantified in bar graphs **(M, N)**. All experiments were repeated with the average values shown. Error bars indicate standard deviations. Two-tailed test was performed for statistical analysis in panels **(M)** and **(N)**, P values; * <0.05, ** <0.01.

**Figure 3:**
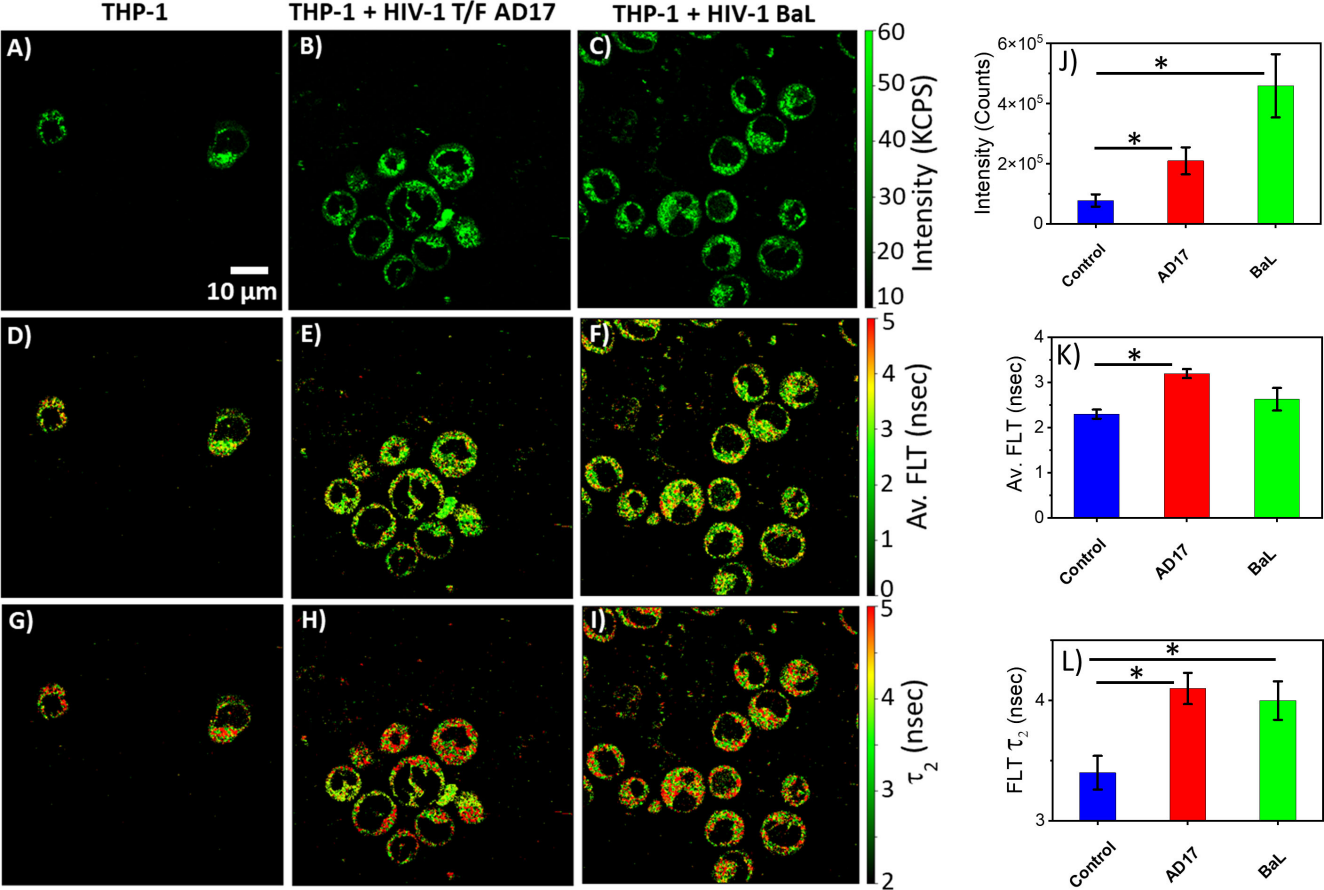
Virion-specific differences in NADH fluorescence intensity and lifetimes. Label-free live 2p-FLIM imaging of NADH with THP-1 cells upon infection with HIV-1 transmitted founder AD17, BaL virions. Differences in NADH fluorescence intensity **(A–C)**, average FLT of NADH **(D–F)**, and bound NADH FLT **(G–I)** for THP-1 cells infected with virions compared to THP-1 control. Image intensity, average FLT, and bound form of NADH FLTs are quantified in bar graphs **(J–L)**. All experiments were repeated with the mean values shown. Error bars indicate standard deviations. Two-tailed test was performed for statistical analysis in **(J–L)**, P values; * <0.05.

The monocytic THP-1 cell line has been used to investigate the mechanisms by which HIV-1 infects and replicates in monocytes and macrophages, as well as the factors that influence the latency of HIV-1 in these cells (49, 50). Label-free live 2p-FLIM imaging of NADH in THP-1 cells upon infection with HIV-1 JRFL pseudovirions after 90 mins at different virion concentrations are illustrated in **Figure 2** (A-C: NADH intensity, D-F: average FLT and G-I: τ_2_, bound form of NADH FLT). **Figure 2** display the time-resolved intensity decays of NADH from control THP-1 (panels J) and HIV JRFL pseudovirus (p24 values of 6µg/ml and 12µg/ml) infected THP-1 cells (panels K and L). A significant change in NADH intensity and FLT in the bound form of NADH in THP-1 cells upon incubation with JRFL compared to THP-1 control is observed and quantified in bar graphs (**Figures 2M, N**). It is important to note that the FLT is independent of probe (NADH in this case) concentration, so any FLT changes observed in the bound form of NADH (τ_2_) represent the inherent changes in molecular properties. To further investigate if the changes in NADH levels in infected cells are associated with multiple rounds of infections, we have used T/F AD17 and BaL-IMC infected THP-1 cells as shown in (**Figures 3A–C**: NADH intensity, D-F: average FLT and G-I: τ_2_ bound form of NADH FLT). Using a bi-exponential fit (see methods), the intensity decay of NADH from uninfected THP-1 cells yielded a τ_1_ value of 0.8 nsec (f_1_ = 0.58) and τ_2_ value of 3.2 nsec (f_2_ = 0.42) with a χ^2^ value of 1.19. In contrast, the bi-exponential fit with the intensity decay of NADH from HIV-1 JRFL infected THP-1 cells exhibited a τ_1_ value of 1.0 nsec (f_1_ = 0.27) and τ_2_ value of 4.25 nsec (f_2_ = 0.73) with a χ^2^ value of 1.16. Likewise, the fit to the intensity decay of NADH from HIV-1 AD17 infected THP-1 cells exhibited a τ_1_ value of 0.95 nsec (f_1_ = 0.32) and τ_2_ value of 4.1 nsec (f_2_ = 0.68) with a χ^2^ value of 0.96. Similarly, the two-component fit to the intensity decay of NADH from HIV-1 BaL-IMC infected THP-1 cells displayed a τ_1_ value of 0.98 nsec (f_1_ = 0.33) and τ_2_ value of 4.25 nsec (f_2_ = 0.67) with a χ^2^ value of 1.10. The χ^2^ values for all the fits to the different intensity decay data sets were in the range of 0.96 to 1.19, indicating overall good fit to the experimental data. Interestingly, more HIV-1 infected THP-1 cells were attached to the poly-lysine coated coverslips compared to the uninfected THP-1 cells. Similar to JRFL-infected THP-1 cells, the enhanced fluorescence intensity of NADH and long FLT associated with the bound form of NADH was observed for both BaL and AD17 virion-infected THP-1 cells compared to uninfected THP-1 cells as displayed in **Figure 3**. The two-tailed t-test showed a significant difference in both intensity and FLT of NADH between infected and uninfected cells (**Figures 3J–L**). Similarly, we observed enhanced fluorescence intensity of NADH and longer FLT signaling bound form of NADH on HIV-1 BaL infected T lymphoblast MOLT-4/CCR5 cells (not shown). These experiments revealed how the virus phenotypes and doses affect cellular NADH levels.

**Figure 4:**
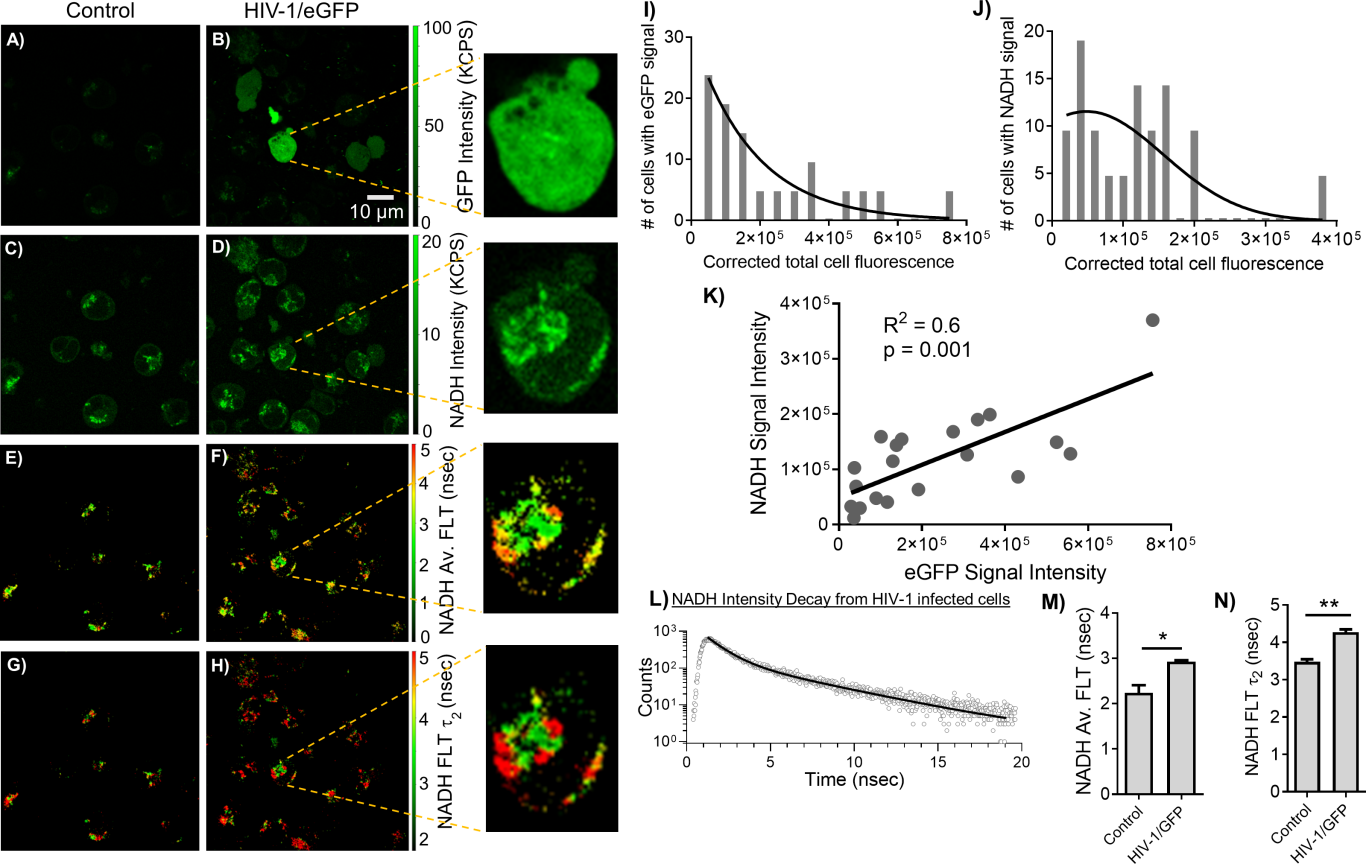
Correlating changes in NADH and eGFP expressing HIV-1 infection. 2p-FLIM imaging of eGFP and NADH with MOLT-4/CCR5 cells upon infection with HIV-1 eGFP virions. Differences in eGFP fluorescence intensity **(A, B)**, NADH fluorescence intensity **(C, D)**, average FLT of NADH **(E, F)**, and bound NADH FLT **(G, H)** for MOLT-4/CCR5 cells infected with virions compared to MOLT-4/CCR5 control. Close-up views of infected cells are shown in the panels adjacent to (B, D, F, H). Intensity histograms from corresponding images from cells with eGFP **(I)** and NADH **(J)** are shown. **(K)** shows the correlation plot between eGFP and NADH from individual cells. **(L)** shows the intensity decay of NADH from HIV-1 infected cells. The dots represent the experimental data and solid line is the bi-exponential fit to the intensity decay (see methods). Average FLT and bound form of NADH FLTs are quantified in bar graphs **(M, N)**. All experiments were repeated, with the average values shown. Error bars indicate standard deviations. Two-tailed test was performed for statistical analysis in panels **(M)** and **(N)**, P values; * <0.05, ** <0.01.

The MOLT-4/CCR5 cells are highly permissive for infection by HIV-1. A recent study (51) reported that HIV-1 selectively infects metabolically active CD4+ T cells with high oxidative phosphorylation and glycolysis. Blocking metabolic pathways in HIV-1 infected cells can stop the virus replication process early on and kill the cells. This suggests that HIV-1 is vulnerable to metabolic disruptions, which could be exploited for therapeutic purposes. Accordingly, we have set out to identify and evaluate the relationship between HIV-1 infection and altered metabolism using an indicator of metabolic activity (NADH levels) and MOLT-4/CCR5 cells during HIV-1 infection. Toward this objective, eGFP expressing HIV-1 BaL was incorporated to assess HIV-1 infection correlated with NADH levels. The MOLT-4/CCR5 cells showed a positive eGFP signal upon being infected with eGFP expressing HIV-1 BaL pseudovirus (**Figure 4B**). **Figures 4D, F, H** showed a noticeable increase in the level of NADH intensity, average FLT of NADH, and longer FLT corresponding to the bound form of NADH for the HIV-1/eGFP infected MOLT-4/CCR5 cells at 48 hours post-infection. Next, HIV-1/eGFP infected MOLT-4/CCR5 cells expressing eGFP fluorescence were analyzed using ImageJ and compared with the corresponding NADH signal. (**Figures 4I, J**). A positive correlation between eGFP and NADH fluorescence is observed, indicating that higher infection of MOLT-4/CCR5 cells leads to an increased level of NADH signal (**Figure 4K**). The intensity decay of NADH from HIV infected cells is shown in **Figure 4L**. A bi-exponential fit was used to analyze the intensity decay, which yielded two decay times: 0.9 nsec (32%) and 4.1 nsec (68%). The fit had a χ^2^ value of 1.06, indicating a good fit to the data. In contrast, a mono-exponential fit to the intensity decay of eGFP signal from the HIV infected cell recorded using a separate detection channel (see methods) exhibited a fluorescent lifetime of 2.6 nsec with a χ^2^ value of 1.29. A single fluorescence lifetime of 2.6 nsec observed for eGFP expressed THP-1 cells detected in the 500 to 529 nm spectral window (which is the optimal emission spectral region for eGFP) confirms that the fluorescence signals emanated from eGFP molecules (15). The changes in NADH’s average FLT and NADH’s bound form for the HIV-1/eGFP infected MOLT-4/CCR5 cells compared to uninfected cells are quantitated **(** **Figures 4M, N** **).**

### 2p-FLIM defines HIV-1 infection profile in tissues

**Figure 5:**
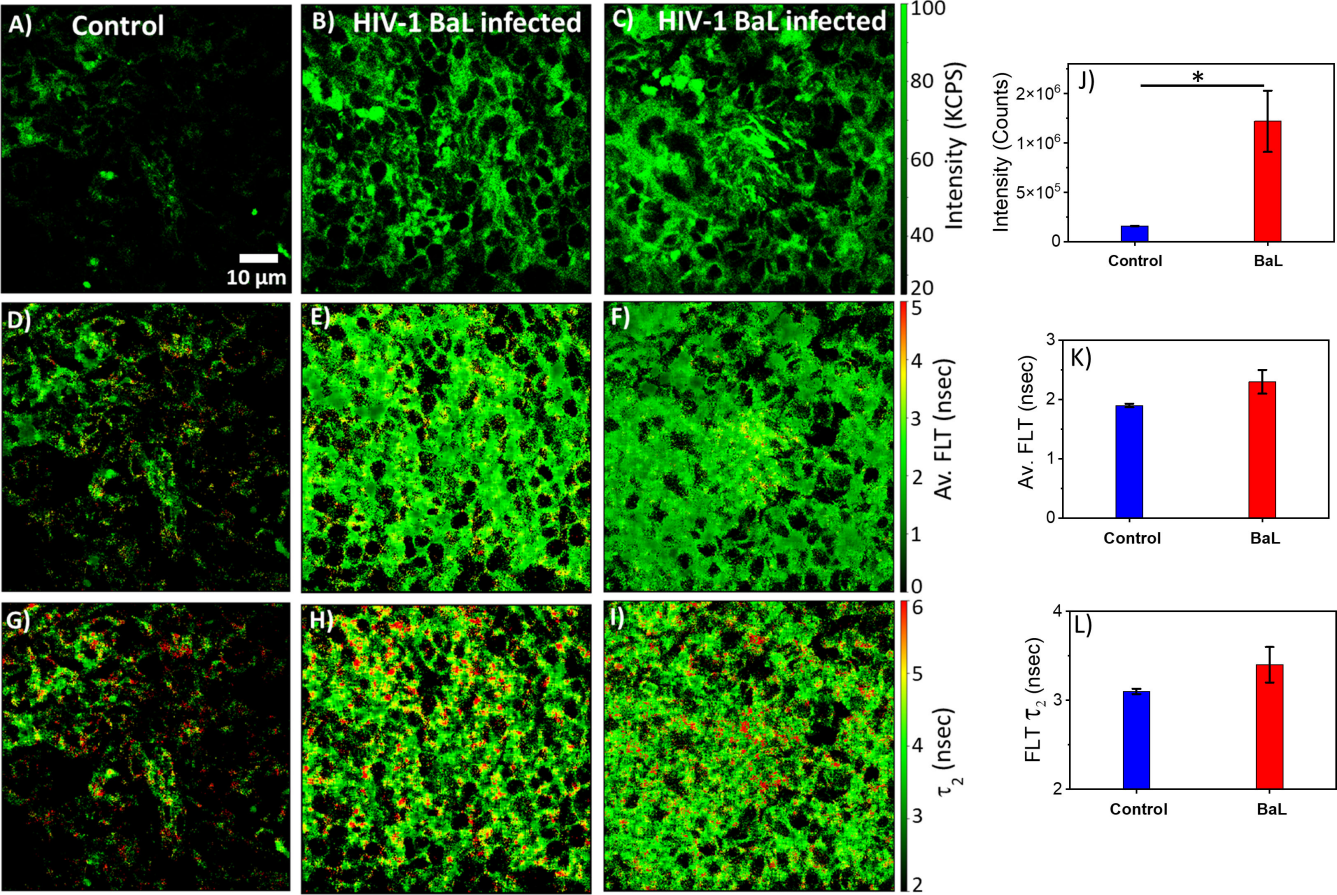
2p-FLIM identifies differences in HIV-1 infected spleen. Label-free 2p-FLIM measurements with humanized NSG mouse spleen tissue section of 6 µm thick. Un-infected control (left column), HIV BaL infected mouse for six months (middle and right columns). **(A–C)**: NADH intensity; Middle **(D–F)**: average FLT; **(G–I)**: bound NADH FLT. Triplicate measurements from different areas were performed. Representative images are shown. Image intensity, average FLT, and bound form of NADH FLTs are quantified in bar graphs **(J–L)**. Two-tailed test was performed for statistical analysis in **(J–L)**, P values; * <0.05.

**Figure 6:**
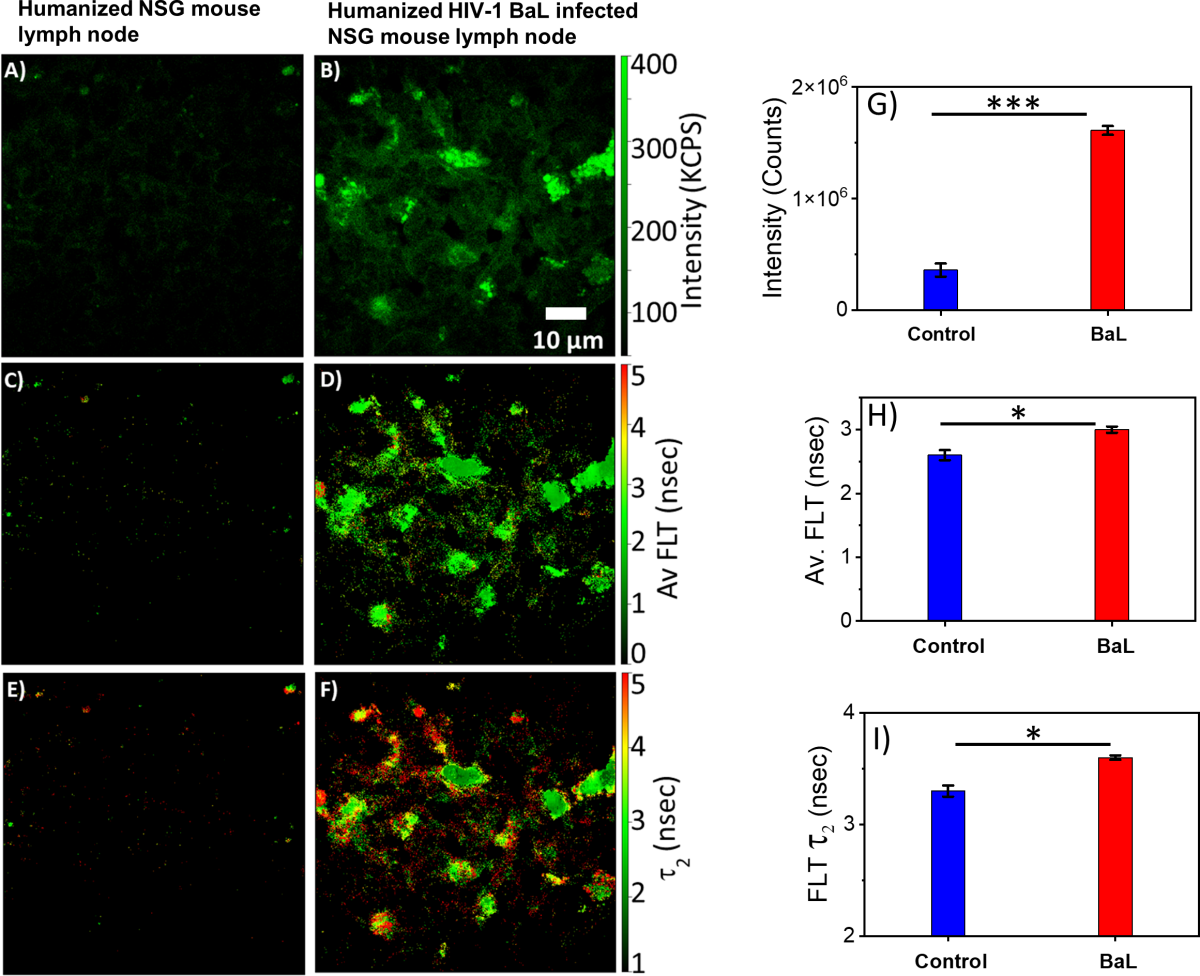
2p-FLIM characterizes differences in an HIV-1 infected lymph node. Label-free 2p-FLIM imaging of NADH with uninfected (left panels) and HIV BaL virus-infected (right panels) NSG mouse lymph node tissue section of 7 µm thick. **(A, B)** Intensity; **(C, D)** Average FLT; **(E, F)** bound NADH FLT. Triplicate measurements from different areas were performed. Representative images are shown. Image intensity, average FLT, and bound form of NADH FLTs are quantified in bar graphs **(G–I)**. Two-tailed test was performed for statistical analysis in **(G–I)**, P values; * <0.05, ** <0.01, *** <0.001.

HIV-1 utilizes metabolically active cellular environments for establishing productive and latent HIV-1 infection in CD4+ T cells (51). Consequently, it is important to identify infected cells and tissues to study the relationship between metabolic activity and T cells in different tissues where HIV-1 replicates. Towards this goal, 2p-FLIM was applied to extract NADH binding profiles within infected tissues from spleen and lymph nodes of humanized NSG mice. 2p-FLIM studies with cryo-sectioned tissues from HIV-1 BaL-infected humanized NSG mice (39) are presented (**Figures 5**, **6**). These experiments also include control spleen and lymph node sections. For each FLIM measurement with the HIV-infected tissue samples, we used a corresponding control uninfected mouse tissue section sample detected under identical settings (same excitation wavelength, incident laser power, detection emission filter, and image acquisition parameters). Label-free 2p-FLIM measurements with humanized NSG mouse spleen tissue display a substantially lower NADH level than the HIV-1 BaL-infected NSG mouse (**Figures 5A–C**). The average FLT and bound form of NADH FLT showed a longer lifetime with a higher fractional amount for the infected tissues, implying a consistent qualitative and quantitative approach to distinguish between uninfected versus HIV-1 infected tissues. The significant difference in NADH signal level is more evident for the HIV-1 BaL infected lymph node tissue section (**Figures 6B, G**). Quantitative analyses evaluating changes in NADH intensity and FLT are presented for infected versus control tissue samples. The two-tailed t-test showed a significant difference in both intensity and FLT of NADH between infected and control tissue sections (**Figures 6G–I**). A likely explanation of the significantly higher levels of NADH including the protein bound form of NADH observed in the infected lymph nodes compared to uninfected control is due to the fact that HIV-1 virions is harbored at these sites. This is a noteworthy observation from this study. A two-tailed test was performed for statistical analysis in panels (G, H & I) of **Figure 6** yielded P values of * <0.05, and *** <0.001.

### 2p-FLIM correlates HIV-1 infection and metabolic disruption

**Figure 7:**
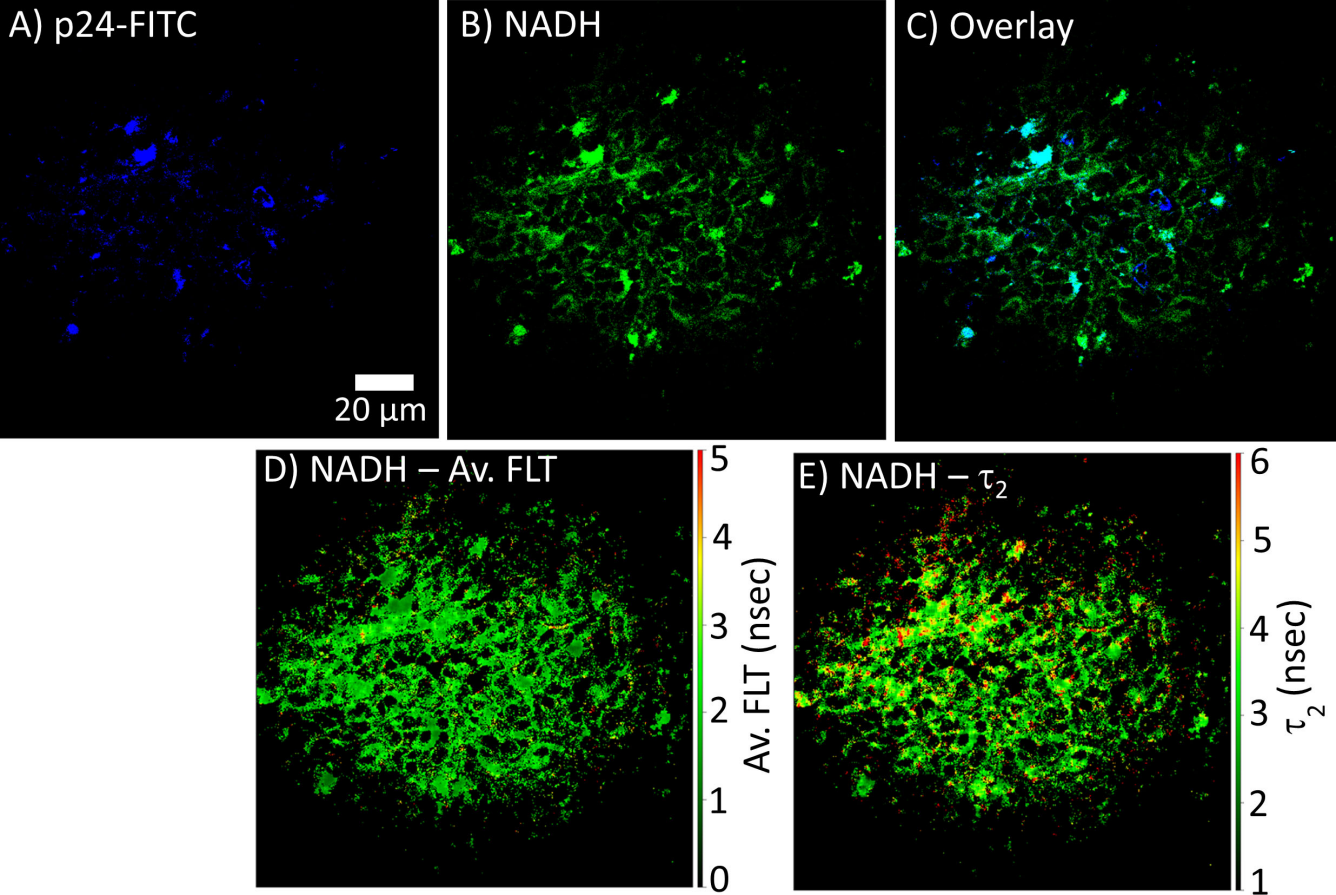
2p-FLIM identifies metabolic differences in HIV-1 infected tissue. Fluorescence intensity images of **(A)** p24-FITC and **(B)** NADH with HIV BaL infected NSG mouse lymph node tissue section of 7µm thick by 2p-FLIM. Panel **(C)** shows the overlay of p24-FITC and NADH fluorescence. **(D, E)** show NADH’s average FLT and NADH’s bound form (τ_2_), respectively.

**Figure 8:**
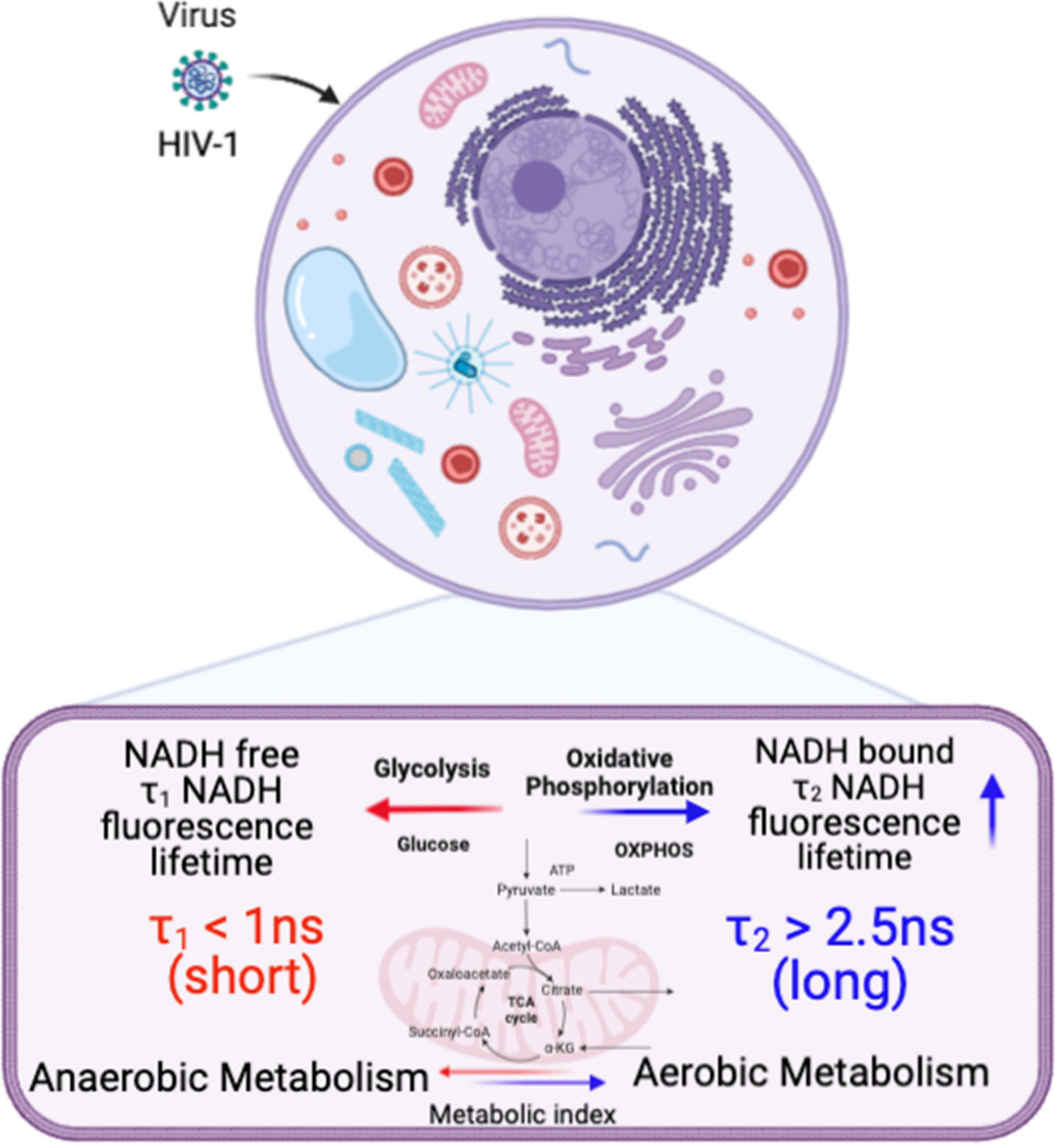
A schematic illustration of cellular metabolism in HIV-1 infected cells and tissues by spatiotemporal imaging using Biorender (see discussion).

FLIM can be used to determine whether there are differences in NADH signal distribution in tissues (lymph node and spleen) in control versus HIV-1 BaL infected mice. The method is also useful to establish the correlation between infection sites versus metabolic distribution around the infectious sites. For determining the infection sites in the tissue, the antigen in the infected cryo-sectioned tissues is detected by a FITC-labeled p24 detection antibody. Our 2p-FLIM setup allows for the simultaneous detection of FITC and NADH in two different detection channels with a single excitation wavelength at 780nm and detection with an appropriate dichroic, short pass, and band pass filters. An example of two-photon imaging of p24-FITC and NADH with HIV-1 BaL infected NSG mouse lymph node (cells in the mesenteric lymph nodes of CD34+ hematopoietic stem cell reconstituted mice) tissue is shown in **Figure 7**. Consequently, it creates a microscopic framework for contextualizing the “snapshot” tissue images of localized infection with metabolic information, as can be visualized in **Figures 7A** (p24-FITC), **7B** (NADH intensity), and **7C** (merged intensity images of p24-FITC and NADH). Thus, 2p-FLIM can be used to determine the correlation between the infection locus and the overall change in metabolic signature *via* NADH. **Figures 7D, E** represent NADH’s average FLT and NADH’s longer FLT (bound form) in the HIV-1 infected mouse lymph node tissue. The substantial increase and fraction of longer FLT of NADH correspond with an enzyme-bound fraction is consistent with an increase in oxidative phosphorylation (OxPhos) (4, 52, 53). Indeed, CD4+ T cells with elevated oxidative phosphorylation are selectively infected by HIV-1 (51). Furthermore, the label-free quantitation of endogenous NADH fluorescence by high-spatial and temporal imaging of virally infected systems at single organism, sub-cellular, cellular, and tissue levels provides a mechanistic view of the microbial-host interface involving perturbations in cellular metabolism for future studies (**Figure 8**). The ability to determine discrete and quantitative changes of NADH lifetimes, label-free and with minimal invasion in cells and tissues, presents an excellent opportunity to evaluate virally induced shifts in metabolism throughout the infection life cycle.

## Discussion

HIV-1 adapts to the host’s metabolic environment in order to complete its replication cycle. This adaptation may help the virus evade the immune system by making it difficult for the immune system to detect and attack the virus. Interestingly, the close relationship between HIV-1 infection and cell metabolism can be used to develop new treatments that impair HIV-1 replication (51). To better understand how cellular processes are affected by HIV-1 infection, we have employed a label-free 2p-FLIM method for identifying, characterizing, and quantitating HIV-1-induced metabolic changes in cells and tissues. The intrinsic fluorescence of NADH used by 2p-FLIM provides a distinctive label-free spectral window for monitoring shifts in the metabolism of HIV-1 infection systems (54). Experiments using multiple viral phenotypes, doses, and incubation times reveal how the virus phenotypes and amounts affect cellular NADH levels. Increases in the NADH levels of infected tissues mirror what we observe in an *in vitro* infection system. Moreover, the label-free quantitation of endogenous NADH fluorescence by high-spatial and temporal imaging of virally infected systems at single organism, sub-cellular, cellular, and tissue level could provide a mechanistic view of the microbial-host interface involving perturbations in cellular metabolism for future studies.

Both fluorescence intensity and lifetime based NADH analyses show the dependence of the number of virus particles (p24) in cells with the changes in NADH fluorescence (intensity and FLT), providing important information about the changes in NADH metabolism corresponding with the relative amounts of virus particles (**Figure 4**). FLT is an inherent parameter that depends on the molecular environment of the fluorescent probe (NADH free or bound) and is independent of the probe concentration, excitation intensity, and light scattering. Thus, fluorescence decay rates can reflect the interactions of a molecular probe with its biological environment. Consequently, the NADH fluorescence decay rates quantified within tissues reflect the extent to which a probe (NADH) is either free or bound to a target. Moreover, 2p-FLIM mitigates background signals for tissue imaging as the fluorescent lifetime of the NADH can be differentiated from non-specific fluorescence. This is identified by differences in FLTs for p24 confirmed HIV-1 infected tissues (**Figure 7**). Accordingly, the presented label-free approach allows for comparing different populations of cellular and subcellular features regardless of the concentration of NADH and, more importantly, can be correlated between the relative amounts of viruses in cryo-sectioned tissues in comparison to the NADH signal level. Our results indicate 2p-FLIM’s sensitivity in assessing and quantitating acutely infected cells and their cell-mediated effects on nearby surrounding cells and tissue.

Label-free live 2p-FLIM imaging of cells exposed to infectious virus particles can potentially establish temporal kinetics of epitope exposure during infection. Accordingly, 2p-FLIM could be applied to reveal conditions of epitope presentations *via* single cells versus cell-cell contacts or cell clustering events. Using FLTs, these studies also allow for comparing subcellular features of different populations of single cells independent of the concentration of NADH in each cell. The relative amount of virus internalized per cell is linked with the NADH signal level (intensity and FLT), thus establishing a correlation of viral infection with associated changes in cellular metabolism, as measured by NADH fluorescence. Indeed, one particularly exciting application of 2p-FLIM NADH analysis is the ability to identify differences in the metabolic state of virally infected (p24 positive) cells and their effect on neighboring non-infected (p24 negative) cells and tissue by quantitating differences in NADH fluorescence intensities and lifetimes. Our experiments indicate increased aerobic metabolism for HIV-1 infected cells, tissues, and close neighboring cells. This increase is illustrated by changes in both free and bound forms of NADH as well as corresponding changes in FLTs. Discrete changes in FLTs can be correlative with changes in the metabolic states of cells and tissues. These studies support a previous observation of HIV-1 infection susceptibility, cell metabolism, and identification of HIV-1 latent reservoirs (51).

We observe significant HIV-1-induced differences in metabolic states as measured by NADH fluorescence intensities and lifetimes for *in vitro* and *in vivo* studies. The higher NADH signal intensity for infected cells or tissues is significant since the bound form of NADH has a 5-times higher quantum yield than the free form of NADH (2, 4, 55). Therefore, discrete cellular NADH intensities and lifetimes associated with oxidative phosphorylation and infection can be quantitated, monitored, and compared with cells exhibiting glycolysis. Since FLIM provides a way to extract different fluorescence lifetimes and contributions for each species using multi-component fitting routine and thereby determining free- versus protein-bound NADH in the present study, free NADH relates to a shift towards glycolysis pathway (4, 7, 52, 54, 56). Furthermore, NADH lifetime is varied significantly depending on the enzyme it is bound with. This has been precisely displayed in a phasor plot (53). Based on this study (53) and others (4, 52), we imply that long FLT observed for NADH in infected cells and tissues is due to the bound form of NADH. Specifically, the long fluorescence lifetimes of NADH observed in the HIV infected cells and tissue section in the current study are due to the bound form of NADH to NOX (NADH oxidase family) enzymes. Related studies reported that inflammatory monocytes, activated microglia, and astrocytes expressing NOX1 as major cellular sources of oxidative stress (57).

In summary, graphical representations of NADH intensities and FLTs provide unique signatures for monitoring, plotting, and comparing cellular and tissue metabolism changes with the longer FLTs (bound NADH) associated with oxidative phosphorylation and shorter FLTs related to glycolysis (free NADH). These studies also provide a framework using label-free 2p-FLIM for quantitating metabolic changes induced by HIV-1 infection. Experiments using multiple viral phenotypes, doses, and incubation times reveal how the virus phenotypes and amounts affect cellular NADH levels. Increases in the NADH levels of infected tissues mirror what we observe in an *in vitro* infection system. The ability to actively monitor and quantitatively track HIV-1-induced metabolic differences throughout infection using 2p-FLIM could provide an excellent label-free detection approach for visualizing and recording acute, chronic, and viral latency throughout an infection cycle. Determining whether observed metabolic differences of neighboring non-p24 cells and tissues by label-free 2p-FLIM will help identify and monitor early, acute, chronic, and latent viral reservoirs will be interesting. Implementing the 2p-FLIM method for label-free quantitative monitoring of microbial infection systems has a distinctive potential to address biomedical research needs and technical problems that occur broadly across multiple biological systems or diseases. Moreover, the label-free quantitation of endogenous NADH fluorescence by high-spatial and temporal imaging of viral infected systems at single organism, sub-cellular, cellular, and tissue level could provide a mechanistic view of the microbial-host interface involving perturbations in cellular metabolism for future studies. A schematic demonstrating the link between NADH fluorescence lifetime and metabolic phenotype in HIV-infected cells and tissues is displayed in **Figure 8**.

## Data availability statement

The raw data supporting the conclusions of this article will be made available by the authors, without undue reservation.

## Ethics statement

The studies involving animals were reviewed and approved by University of Maryland, Baltimore (UMB) Institutional Animal Care and Use Committee (IACUC). Human cord blood CD34+ hematopoietic stem cells to engraft NSG mice were purchased from commercial sources. The project was reviewed by the UMB’s Office of Human Research Ethics, which has determined that this submission does not constitute human subjects research as defined under federal regulations [45 CFR 46.102 (d or f) and 21 CFR 56.102(c)(e)(l)] and does not require IRB approval.

## Author contributions

Conceived the idea and designed the research: GS and KR. Performed experiments: GS, SK and KR. Tissue samples and new reagents: GL. Analyzed the data: GS, SK, GL and KR. Initial draft: KR. Extensive editing: GS. Funding acquisition: KR. All authors critically read, edited, and approved the manuscript.
